# Assessing the quality of AI-generated and physician-written discharge summaries: evaluation of an EHR-integrated tool in a Dutch academic hospital

**DOI:** 10.1016/j.ebiom.2026.106247

**Published:** 2026-04-09

**Authors:** Tarannom Mehri, Tun Nadalini, Anne H. Hoekman, Tom P. van der Laan, Katerina Kagialari, Robert K. Wagner, Job N. Doornberg, Rosanne C. Schoonbeek, Charlotte M.H.H.T. Bootsma-Robroeks, Applied Artificial Intelligence in Healthcare Consortium, M. Aalderink, R. Van den Berg, M.T.P. Besouw, A.V. Biere, F.A.J.A. Bodewes, A.L. Boerboom, M.A.J. Borgdorff, M.H. De Borst, M. Bouhuys, B.R. Brandsema, G.H. Bultema, M.J. Crop, H.P.J. Van der Doef, J.W.J. Donkers, J.M. Douwes, R.A. Feijen, F. Fontanella, B. Foreman, V. Gracchi, I. De Groot, G.B. Halmos, A.A. Van Heerwaarde, F. Van den Heuvel, C. Holzhauer, F.F.A. IJpma, E. Kersten, R.J.H. Knoef, M.C.A. Kramer, S. Krishnapillai, M. Labberté, J.M. Lammers, L.B. De Langen, E. Lensen, W.S. Lexmond, E.T. Liem, E. Loeffen, J. Lorius, C. Lubout, J. Ludwig-Roukema, S. Luiten, D. Meijering, C. Out, S. Palthe, M.T.R. Roofthooft, R. Scheenstra, R.S.B.H. Schreuder, M.L. Schrijvers, P.F. Sinnige, W.J. Van Veen, C.A. Te Velde-Keyzer, K.T. Verbruggen, M. Verheijen, F.P.J. Vernimmen, J. De Vries, W. De Weerd, C.L. Welsink, J.E.J. Woolderink, A.T. Zwart

**Affiliations:** a Department of Health Information Office, Information Management Healthcare, University Medical Centre, Groningen, the Netherlands; b Department of Otolaryngology, Head and Neck Surgery, University Medical Centre, Groningen, the Netherlands; c Department of Trauma Surgery / Orthopaedics, University Medical Centre, Groningen, the Netherlands; d Department of Orthopaedic Surgery, Massachusetts General Hospital, Boston, MA, USA; e Harvard Medical School Orthopaedic Trauma Initiative, Boston, MA, USA; f Department of Orthopaedic Surgery and Sports Medicine, Amsterdam UMC Location University of Amsterdam, Meibergdreef 9, Amsterdam, the Netherlands; g Department of Orthopaedic Trauma, Flinders University Medical Centre, Adelaide, Australia; h Department of Paediatrics, Paediatrics Nephrology, Beatrix Children’s Hospital, University Medical Centre, Groningen, the Netherlands

**Keywords:** Large language models, Electronic health records, Discharge summaries, Clinical documentation, Validation study, Health informatics

## Abstract

**Background:**

Large language models (LLMs) offer potential to reduce administrative burden in clinical care by generating discharge summaries. Most prior evaluations have been limited to drafts, small cohorts, or non-integrated settings. Robust validation of fully automated, EHR-integrated systems in real-world practice is lacking.

**Methods:**

This study was conducted in April 2025 at a Dutch academic hospital. A total of 292 paired discharge summaries from multiple departments were evaluated, each consisting of a physician-written and an LLM-generated version. Summaries were independently assessed by eight blinded clinicians using a 5-point Likert scale across completeness, correctness, and conciseness. Trustworthiness was also scored. Domain and total scores were compared with Wilcoxon signed-rank tests, and interrater reliability was quantified using Gwet’s AC2.

**Findings:**

LLM-generated summaries had lower completeness (4.50 (4.00–5.00) vs 5.00 (4.50–5.00); p < 0.001), similar correctness (5.00 (4.50–5.00) vs 5.00 (4.63–5.00); p = 0.14), and greater conciseness (5.00 (4.50–5.00) vs 4.50 (4.00–5.00); p < 0.001) compared with physician-written summaries. Total scores did not differ (14.00 (13.00–15.00) vs 14.00 (13.00–15.00); p = 0.34). Physician-written summaries were trusted by both reviewers in 279 (95.5%) cases, whereas LLM-generated summaries were trusted in 249 (85.3%) cases, partially trusted in 34 (11.6%), and rejected in 9 (3.1%). Interrater agreement for total scores was high (AC2 0.87, 95% CI 0.83–0.90 for LLM; 0.85, 95% CI 0.81–0.89 for physician).

**Interpretation:**

Discharge summaries generated by an EHR-integrated LLM achieved quality ratings comparable to physician-written documents across multiple specialties, with no difference in total scores. Unlike earlier pilot work, this study demonstrates real-world feasibility of automated LLM use in clinical workflows at scale. With appropriate oversight and specialty-specific refinement, such systems could substantially reduce documentation burden while maintaining discharge summary quality.

**Funding:**

This research did not receive a specific grant from any funding agency in the public, commercial, or not-for-profit sectors.

**Research in context**

**Evidence before this study**

Previous evaluations of large language models (LLMs) for hospital discharge summaries have predominantly been conducted in experimental or single-specialty settings. Prototype systems often demonstrated coherent output but were limited by omissions and hallucinations, and models based on structured data still required physician post-editing. More recent studies have suggested that advanced LLMs may produce discharge summaries of quality comparable to physician-written documentation. However, few evaluations have been conducted within fully integrated electronic health record (EHR) environments, across multiple clinical specialties in routine care, or using blinded paired comparisons against physician-written summaries.

**Added value of this study**

This study provides a large-scale evaluation of an LLM directly integrated into the EHR of a Dutch academic hospital. Across 292 paired discharge summaries from multiple departments, eight blinded clinicians found LLM-generated documents comparable to physician-written summaries in completeness, correctness, and conciseness. Interrater agreement was high, and performance was consistent across specialties despite reliance on a single generic prompt. Importantly, no physician post-editing was required.

**Implications of all the available evidence**

The findings suggest that LLMs can generate discharge summaries of quality comparable to physician summaries across several departments. When considered alongside previous experimental studies, this work supports the potential feasibility of automated summary generation to reduce documentation burden. Future implementation research should address long-term safety, workflow effects, educational implications for junior clinicians, and the establishment of robust evaluation frameworks to ensure sustained quality and trust.

## Introduction

The digitalisation of clinical documentation through Electronic Health Records (EHRs) has unintentionally intensified the administrative workload for physicians, raising concerns about its impact on care quality, efficiency, and physician well-being.^1^, ^2^, ^3^ Among the most clinically significant forms of EHR documentation are hospital discharge summaries, which serve as a key communication tool during transitions of care.

A well-prepared discharge summary communicates diagnoses, treatment course, and follow-up plans to outpatient providers and is essential for continuity of care. Prior studies demonstrated that discharge letters of higher quality (i.e., better clarity, completeness, and medical accuracy) were associated with fewer post-discharge complications,^4^ whereas delayed or inadequate summaries increase the risk of information gaps, medication errors, and preventable readmissions.^5^ Despite their importance, discharge summaries are often incomplete or inconsistent, in part due to time constraints and lack of standardisation.^5^ Improving both the efficiency and quality of discharge documentation is therefore a key priority to support safe and timely care transitions while reducing physician burden.

Generative large language models (LLMs) have emerged as a potential solution to reduce the documentation burden in healthcare.^6^ These models can synthesise coherent clinical narratives from structured input data. Initial studies have demonstrated their ability to generate discharge summaries and operative reports within seconds, with content often judged by experts as accurate and clinically useful.^7^ However, most evaluations have been conducted in English-language prototype settings, with limited real-world integration or validation in routine care workflows, especially in non-English contexts.

This validation study examined whether an LLM integrated into the electronic health record can generate Dutch hospital discharge summaries that match the quality of physician-written versions. A total of 292 summary pairs from multiple clinical departments were compared using blinded, paired assessments by trained clinicians. Summaries were rated across three domains, completeness, correctness, and conciseness, using a 5-point Likert scale. It was hypothesised that the LLM-generated summaries would be clinically equivalent in quality, offering a scalable approach to reduce documentation burden without compromising communication standards.

## Methods

### Study design and setting

**Fig. 1:**
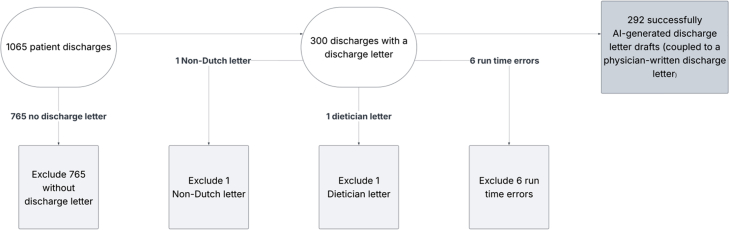
Flowchart of discharge summary selection.

This validation study was conducted in 2025 at a single academic hospital in the Netherlands with approximately 1300 inpatient beds. Cases were drawn from 16 clinical departments (Fig. 1).

### Data sources and case selection

Cases were eligible if a finalised physician-written discharge letter created during a one-week period in April 2025 was available; and if structured clinical data for the same case was available to generate a LLM-based summary. Discharge letters were excluded if there was no finalised physician version, if the letter was written in any language other than Dutch, or if it originated from a non-discharge context (e.g., dietary consultation). Of approximately 1000 discharge cases during the one-week period, 292 met inclusion criteria and were included for evaluation (Fig. 1).

### Model prompting and generation

LLM-generated hospital course summaries were generated with the Drafted Hospital Course tool developed by Epic Systems Corporation, using GPT-4o (OpenAI, San Francisco, CA) deployed through Microsoft Azure OpenAI Service, with a maximum context window of 128,000 tokens and a maximum output length of 4096 tokens. The specific model was selected and maintained by the EHR vendor and could not be configured or substituted by the research team within the in-hospital system.

The prompt used clinical documentation extracted from the electronic health record. All model inputs were derived exclusively from the hospital’s EHR (Epic Chronicles database) and consisted of structured demographic data (including administrative sex as recorded in the EHR), the documented principal problem, problem list entries, coded clinical events, and predefined clinical note types with associated metadata (author, cosigner, provider role, service, and date of service), assembled in chronological order. Information on gender identity was not available in the EHR and was therefore not included.

More granular clinical data elements, including vital signs, laboratory results, imaging findings, operative details, and flowsheet information, were not provided as separate structured inputs and were available to the model only when documented within the clinical notes. Any pre-existing hospital course summary note was intentionally withheld to evaluate the model’s ability to summarise independently.

A standardised prompt template was used with high-level instructions on narrative flow and expected clinical detail. The exact prompt text is proprietary to the EHR vendor and cannot be disclosed verbatim. Because of these proprietary constraints, prompt transparency is inherently limited; however, a structured high-level functional description of the prompt components and their intended roles is provided to support reproducibility.

The prompt was designed to define the task boundaries and intended clinical audience of the hospital course summary, specify structural and formatting expectations, describe how input documentation was organised, and guide the model to prioritise clinically relevant inpatient information while omitting routine or non-critical details. Explicit safeguards were incorporated to discourage unsupported inferences and prevent extrapolation beyond the provided documentation.

Prompt development was iterative and conducted by the vendor to align model behaviour with established clinical documentation standards. Refinements were informed by internal evaluation processes and clinician feedback. The overall structure and intent of the prompt remained stable during the study period, and the same standardised prompt was used across all cases rather than being adapted per specialty or patient. None of the discharge summaries included in this evaluation were used in prompt development or optimisation.

The model was instructed to output the summary in Dutch as the hospital course section of the discharge summary. GPT-4o had a training data cutoff of October 2023 and had no capability to access external systems, internet resources, or retrieval-based augmentation during generation. All inputs were restricted to documentation available within the EHR.

Decoding parameters were fixed at a temperature of 0 and top-p of 1. Each discharge summary was generated once using these fixed parameters. No repeated sampling or alternative generations were performed, and no variability or stochastic sensitivity analyses were conducted. The reported evaluation therefore reflects a single deterministic output per case.

Summaries were generated in separate processes with access to only a single patient’s data, thereby preventing contextual cross-contamination between cases. In the majority of cases, generation occurred within a single-turn request. Input notes were not truncated or split. In rare instances in which input volume exceeded the limit of a single request, sequential requests were used without omitting source material.

Although the LLM was technically integrated within the electronic health record environment at the time of evaluation, it was not deployed for routine clinical use and was activated exclusively for this study. Consequently, clinician raters had no prior exposure to the tool.

This configuration differs from the current clinical deployment, in which LLM-generated discharge summaries are presented to physicians as editable drafts within the EHR. To support transparent interpretation and generalisability of the findings, a comparison between the study-specific configuration and the live EHR implementation is provided in Table 1, including automation level, editability, and intended clinical use.

**Table 1:** Comparison of study-specific configuration and live EHR deployment of the LLM-based discharge summary tool.

System characteristic	Study-specific configuration (this evaluation)	Live EHR implementation (routine clinical use)
EHR integration	Fully integrated within the hospital EHR	Fully integrated within the hospital EHR
Activation status	Activated exclusively for this validation study	Available for routine clinical use
Level of automation	Fully automated	Fully automated
Physician post-editing	Disabled	Enabled
User interaction	No interaction during generation or evaluation	Clinicians are obliged to review and edit generated text
Purpose of use	Isolate and validate intrinsic model output quality	Support clinical documentation in daily workflow
Output handling	Evaluated exactly as generated	Final content determined by physician after editing
Intended evaluation focus	Clinical quality of standalone LLM output	Workflow efficiency, usability, and final documentation quality

### Evaluation framework

Each physician-written and LLM-generated discharge summary pair was independently rated by two clinicians selected from a pool of eight reviewers, with the 292 cases distributed evenly into four subsets. The reviewer pool included four junior and four senior clinicians. Junior physicians were defined as having fewer than five years of post-graduate clinical experience. Senior physicians were defined as having more than five years of clinical experience, including physicians in the final phase of residency and attending physicians.

Prior to the evaluation, all raters received standardised written instructions outlining the study objectives and the definitions of the evaluation domains (completeness, correctness, conciseness, and trust) (Supplementary Fig. S6). No additional calibration or consensus training sessions were conducted.

Raters were blinded to the source of each summary and the order of presentation (LLM-generated vs. physician-written) within each pair was randomised. Reviewers assessed summaries across three domains: completeness (inclusion of all clinically relevant events and decisions), correctness (accuracy and factual consistency), and conciseness (clarity and focus without unnecessary detail or redundancy). The domain definitions were derived from the work of Van Veen et al. (2024) with modifications and rating anchors defined by the research team (Supplementary Fig. S6). A 5-point Likert scale (1 = poor, 5 = excellent) was used for each domain. In addition, reviewers indicated whether they would consider each summary trustworthy for use in clinical care. Trustworthiness was defined as the clinician’s overall confidence in relying on the discharge summary for safe clinical handover and routine clinical use, assessed as a binary judgement without a structured rubric to reflect naturalistic clinical decision-making (yes/no). Raters were asked the following question: “Would you trust this discharge summary for routine clinical use?”. Clinically relevant omissions were captured within the completeness domain. In addition to Likert-scale ratings, raters were able to provide free-text comments during the evaluation. Raters were explicitly instructed to pay attention to hallucinations and clinically relevant omissions.

Differences in domain scores were categorised with a difference of ≥3 points indicating large advantage or disadvantage for the LLM-generated summary, a difference of 1–2 points a moderate advantage or disadvantage, and a difference of 0 being equivalent. Overall quality was evaluated by summing domain scores (range 3–15). Thresholds for interpreting these differences were predefined. For total scores, differences in scores were categorised with a difference of ≥4 points indicating large advantage or disadvantage for the LLM-generated summary, a difference of 2–4 points a moderate advantage or disadvantage, and a difference of <2 being equivalent.

Additionally, a sub analysis was performed to examine LLM-generated summaries that received a completeness score of ≤2 points (out of 5). For these cases, missing components were identified and categorised into clinical course, management plan, diagnostics, diagnosis, treatment, medication, and medical history.

The Supplementary Materials contain PHI-free examples illustrating the structure of both physician-written and LLM-generated discharge summaries (Supplementary Figs. S4 and S5), along with the instructions provided to raters during the evaluations (Supplementary Fig. S6).

### Statistical analysis

Descriptive statistics were used to summarise overall domain scores and departmental distributions. Domain ratings did not follow a normal distribution, scores were reported as medians. Paired LLM-generated and physician-written summaries were compared using Wilcoxon signed-rank tests. Trust ratings were compared using McNemar’s test, as trust was evaluated by the same reviewers for both versions of each summary. Inter-rater reliability for domain scores was assessed using Gwet’s AC2 due to the skewed ordinal data and subset design. Gwet’s AC2 values with 95% Confidence Intervals were calculated per domain and for the total score. Statistical analyses were conducted using SPSS® Statistics version 28.0 (IBM Corp., Armonk, NY, USA) and R version 4.4.1 (R Foundation for Statistical Computing, Vienna, Austria) using the irrCAC package (v1.0).

### Reporting standards

This study is reported in accordance with the DECIDE-AI reporting guideline for clinical artificial intelligence evaluation studies. The completed checklist is provided as Supplementary Material.

### Ethical considerations

This study was conducted in accordance with the Declaration of Helsinki and approved by the institutional ethics review board (reference: M24.328217). The institutional review board granted a waiver of informed consent because the study involved retrospective evaluation of routinely collected documentation data and did not involve patient contact or alteration of clinical care. The research protocol was prospectively registered in the institutional research registry of the University Medical Centre Groningen (Panama system; registration number 19035) on 1 November 2023. This registry is maintained internally and is not publicly accessible. All LLM interactions were managed within the EHR environment under existing contractual agreements between the hospital and the EHR vendor. Human review of model inputs or outputs by external third parties (including cloud service providers) was disabled. All patient-sensitive data were encrypted in transit. Decrypted inputs and model outputs remained within the hospital’s secure digital infrastructure and were not accessible to external parties. The AI system used (GPT-4o) was deployed as a documentation support tool and not for diagnostic or therapeutic decision-making, and thus does not qualify as a medical device under current European regulations. All generated summaries were stored securely within the hospital’s internal infrastructure. No patient-identifiable data were shared with external vendors, including the EHR provider or OpenAI. Data privacy officers were actively involved throughout the study to ensure compliance with applicable data protection laws, including the General Data Protection Regulation (GDPR).

### Role of funders

This study did not receive specific funding from any public, commercial, or not-for-profit agency. The EHR-integrated AI tool was developed and maintained by the commercial vendor as part of routine system functionality; however, the vendor had no role in study design, case selection, data extraction, statistical analysis, interpretation of results, or preparation of the manuscript. The research team independently conducted the evaluation and retained full access to the study data. No patient-identifiable data were shared with the vendor.

## Results

### Study sample and distribution

**Fig. 2:**
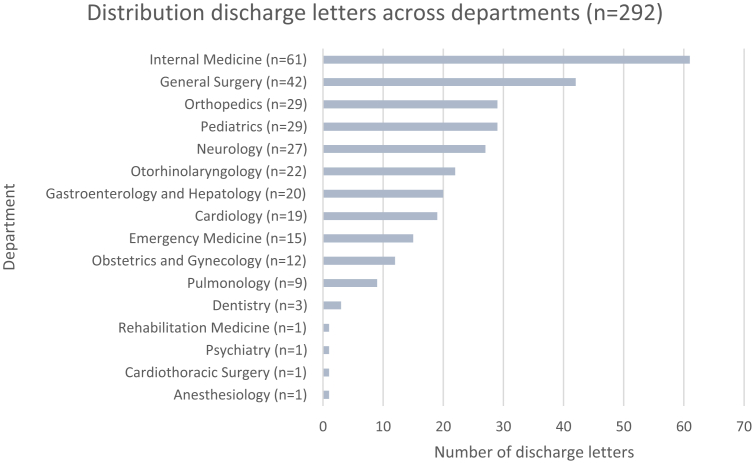
Distribution of discharge letters across departments (n = 292).

From April 14 to 20, 2025, a total of 1065 patients were discharged from the hospital. Of these, 300 had a finalised physician-written discharge letter. Eight cases were excluded; specifically due to generation failures (n = 6), non-discharge contexts (n = 1), or documentation in a non-Dutch language (n = 1), resulting in 292 discharge summary pairs being analysed (Fig. 1). Each pair consisted of one physician-written and one LLM–generated summary. Discharge summaries were from a wide range of departments, including internal medicine (n = 63), general surgery (n = 45), cardiology (n = 19), and neurology (n = 28). Discharge summaries originated from 16 clinical departments, which are shown in Fig. 2; department-level analyses were restricted to departments with ≥2 cases. Interrater agreement for the overall score was a Gwet’s AC2 of 0.866 (95% CI (0.835–0.896)) for LLM-generated summaries and a Gwet’s AC2 of 0.851 (95% CI (0.811–0.891)) for physician-written summaries (Table 2).

**Table 2:** Gwet’s AC2 values (95% CI) for interrater agreement across evaluation domains.

Domain	LLM-generated	Physician-written
Completeness	0.74 (0.70–0.80)	0.88 (0.92–0.96)
Correctness	0.93 (0.90–0.95)	0.94 (0.92–0.96)
Conciseness	0.88 (0.85–0.91)	0.86 (0.83–0.90)
Total score	0.87 (0.84–0.90)	0.85 (0.81–0.89)

### Descriptive statistics

Physician-written discharge summaries had a higher mean word count and greater variability in length than LLM-generated summaries. The mean word count of physician-written summaries was 204 (SD 114; range 40–966), compared with 124 (SD 19; range 68–176) for LLM-generated summaries (Supplementary Table S2). Word counts of physician-written summaries showed a right-skewed distribution, whereas LLM-generated summaries were more narrowly distributed. Across departments, physician-written summaries showed wide variation in length, while LLM-generated summaries were relatively consistent (Supplementary Table S3).

### Overall performance

LLM-generated summaries received a median completeness score of 4.5 (4.0–5.0), compared to 5.0 (4.5–5.0) for physician-written summaries (p < 0.001). Correctness scores were 5.0 (4.5–5.0) for LLM summaries and 5.0 (4.6–5.0) for physician summaries (p = 0.14). Conciseness scores were 5.0 (4.5–5.0) for LLM summaries and 4.5 (4.0–5.0) for physician summaries (p < 0.001). The total score was 14.0 (13.0–15.0) for both summary types (p = 0.34) (Table 3).

**Table 3:** Performance metrics by evaluation domain.

Domain	LLM-generated	Physician-written	p-value (Wilcoxon signed-rank test)
Completeness	4.5 (4.0–5.0)	5.0 (4.5–5.0)	<0.001
Correctness	5.0 (4.5–5.0)	5.0 (4.6–5.0)	0.14
Conciseness	5.0 (4.5–5.0)	4.5 (4.0–5.0)	<0.001
Total score	14.0 (13.0–15.0)	14.0 (13.0–15.0)	0.34

Median (IQR) scores for completeness, correctness and conciseness.

### Trust ratings

Physician-written discharge letters were trusted by both reviewers in 279 (95.5%) of 292 cases and by one of two reviewers in 13 (4.5%) cases; none were rejected by both reviewers.

LLM-generated discharge letters were trusted by both reviewers in 249 (85.3%) cases, by one of two reviewers in 34 (11.6%) cases, and rejected by both in 9 (3.1%) cases.

### Domain-level evaluation

**Fig. 3:**
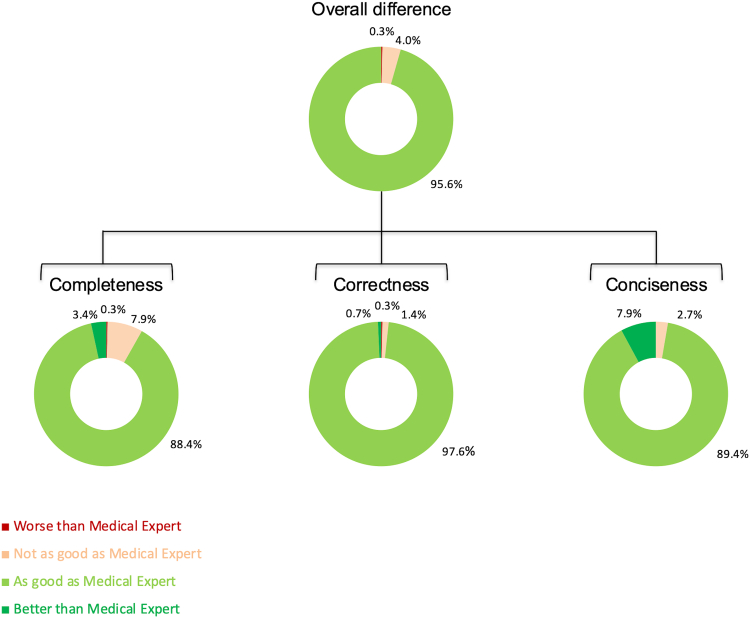
Breakdown of difference in performance per domain.

In domain-specific assessment, LLM-generated letters were rated equivalent to physician summaries in 88.4% for completeness, 97.6% for correctness, and 89.4% for conciseness. Only 0.3% of summaries were rated lower compared to physician-written letters in completeness and correctness, and none for conciseness (Fig. 3). A pairwise comparison revealed that the LLM-generated summary received an equivalent or higher score than the physician-written summary in 91.8% of cases for completeness, 98.3% for correctness, and 97.3% for conciseness (Fig. 3).

### Low-completeness cases (N = 20)

20 LLM-generated summaries (6.8%) received a completeness score of 2 points or less out of 5 possible points. The most commonly omitted components were the clinical course (50%), management plan (45%), and additional diagnostics (35%). Other frequently missing items included the diagnosis or working diagnosis, treatment plans, medication details, and medical history (Table 4).

**Table 4:** Most frequently missing components of completeness in LLM-generated discharge summaries.

Missing Component	Count	Percentage (%)
Clinical course	10	50%
Management plan	9	45%
Additional diagnostics	7	35%
Diagnosis/working diagnosis	5	25%
Treatment	5	25%
Medication	5	25%
Medical history	4	20%
Follow-up/discharge advice	2	10%
Physical examination	1	5%
Lab results	1	5%

### Subgroup analysis by department

**Fig. 4:**
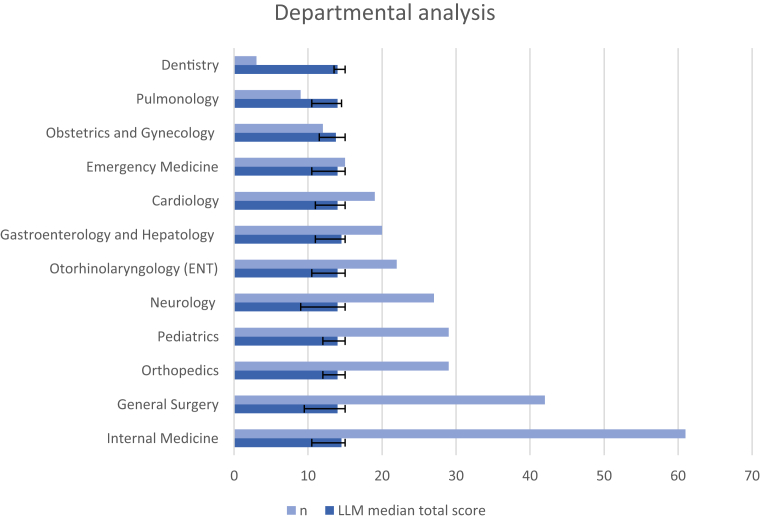
Performance per department, median total score and amount of discharge letters.

**Fig. 5:**
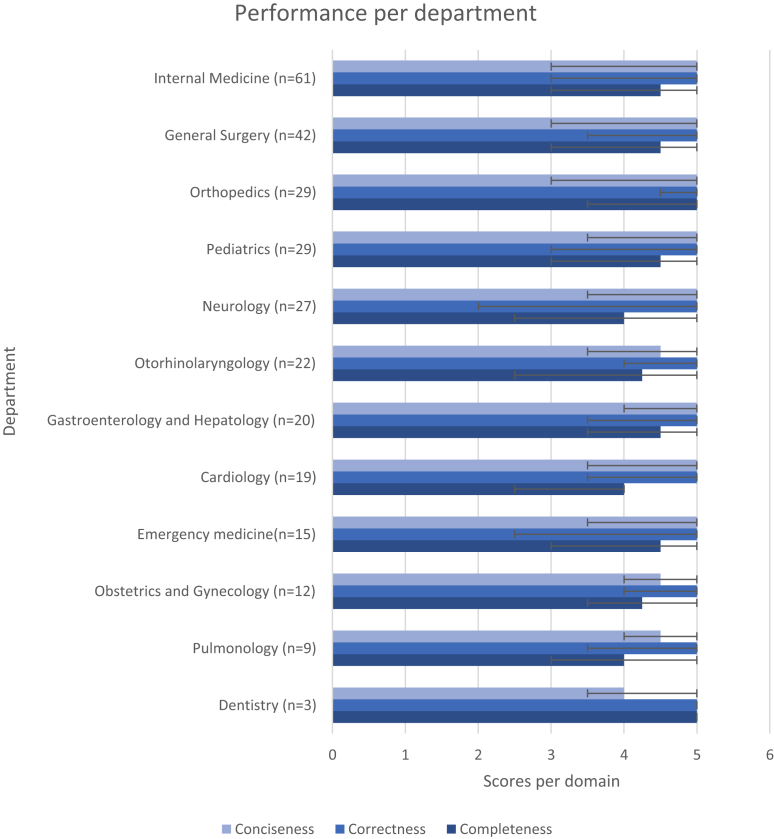
Median (IQR) scores of domain specific performance per department.

To assess the consistency of LLM-generated discharge summary performance across clinical specialties, median domain scores with interquartile ranges (IQRs) were calculated for each department with at least two discharge summaries (n = 12) (Table 5, Fig. 4). Completeness was highest in Orthopaedics (5.0 (3.5–5.0)) and Dentistry (5.0 (5.0–5.0)), while other departments ranged from 4.0 to 4.5. Correctness was uniformly high (5.0 across all departments), but variability differed: in Neurology and Emergency Medicine scores showed wide distributions (2.0–5.0 and 2.5–5.0, respectively), whereas in Dentistry and Orthopaedics variability was limited, with scores concentrated near the maximum (5.0–5.0 and 4.5–5.0). Conciseness reached 5.0 in most departments, with lower values in Dentistry (4.0 (3.5–5.0)) and in Pulmonology, Obstetrics and Gynaecology, and Otorhinolaryngology (all 4.5 (4.0–5.0 or 3.5–5.0)) (Table 5). Although Obstetrics and Gynaecology had the lowest median total score among departments (13.75 (11.50–15.00)), all departmental scores fell within the predefined clinically acceptable range. Detailed departmental scores are provided in Fig. 5.

**Table 5:** Median (IQR) total scores of LLM generated letters per department.

Department	LLM Total Score	N
Dentistry	14.00 (13.50–15.00)	3
Pulmonology	14.00 (10.50–14.50)	9
Obstetrics and Gynaecology	13.75 (11.50–15.00)	12
Emergency medicine	14.00 (10.50–15.00)	15
Cardiology	14.00 (11.00–15.00)	19
Gastroenterology and Hepatology	14.50 (11.00–15.00)	20
Otorhinolaryngology (ENT)	14.00 (10.50–15.00)	22
Neurology	14.00 (9.00–15.00)	27
Orthopaedics	14.00 (12.00–15.00)	29
Pediatrics	14.00 (12.00–15.00)	29
General surgery	14.00 (9.50–15.00)	42
Internal medicine	14.50 (10.50–15.00)	61

## Discussion

This study aimed to evaluate the clinical quality of discharge summaries generated by a generative AI tool that is directly integrated into the electronic health record (EHR) of a Dutch academic hospital. The primary objective was to determine whether LLM-generated summaries are comparable in completeness, correctness, and conciseness to those written by physicians.

Across 292 discharge summary pairs from multiple departments, LLM-generated summaries achieved evaluation scores closely aligned with physician-written letters. While minor differences were observed, favouring physicians in completeness, and the LLM in conciseness, most summaries were rated equivalent. No significant difference was found for correctness or total score. Trust ratings were high for both groups. Physician-written summaries were trusted by both reviewers in 279 (95.5%) cases, and LLM-generated summaries in 249 (85.3%) cases, with partial agreement in 34 (11.6%) and disagreement in only nine (3.1%). Interrater agreement for the overall score was high, with Gwet’s AC2 values of 0.866 for LLM-generated and 0.851 for physician-written summaries, indicating strong consistency between raters and supporting the reliability of these results.

Interpretation of these findings should consider the timing of system deployment. At the time of evaluation, the LLM-based discharge summary tool was not yet deployed in routine clinical practice, and clinicians had no prior exposure to the system. As a result, trust assessments may partly reflect an early-adoption context rather than established clinical familiarity, which should be considered when interpreting trust-related outcomes.

The observed difference between trust ratings and domain-specific quality scores further highlights that perceived trustworthiness and evaluated textual quality are related but distinct constructs. Even under blinded conditions, clinicians may recognise stylistic characteristics commonly associated with LLM-generated text, which can influence trust judgements independently of completeness or correctness. In prior work by our group, clinicians were able to correctly identify LLM-generated discharge summaries in most cases, while trust in LLM-generated and physician-written summaries was comparable.^8^ These findings indicate that recognisability does not necessarily preclude trust. In the present study, although trust ratings for LLM-generated summaries were lower than for physician-written summaries, the absolute difference was modest, with agreement on trust in most cases and disagreement in only a small minority. Together, these observations suggest that trust is shaped not only by textual quality but also by familiarity, expectations, and perceptions of authorship, particularly in early stages of deployment.

Early efforts to generate discharge summaries using large language models (LLMs) often relied on prototype tools outside clinical workflows. Hartman et al. used BERT-based models to generate neurology discharge content, achieving moderate validity but reporting omissions and hallucinations.^9^ Ganzinger et al. developed LLM systems to draft summaries from structured data, but these still required physician post-editing.^10^ Zaretsky et al. applied GPT-4 to translate clinician-authored summaries into patient-friendly language, improving readability while still noting frequent omissions and occasional hallucinations.^11^

Recent evaluations have begun to explore more advanced LLM applications. Williams et al. assessed GPT-4–generated discharge summaries and found them comparable to physician-authored ones in accuracy and quality, while being more concise and coherent.^12^ In emergency medicine, an EHR-integrated study demonstrated that AI-assisted LLM-generated discharge notes, edited by clinicians, substantially reduced documentation time while maintaining high documentation quality.^13^ The present study builds on such work by being among the first to evaluate a fully automated, EHR-integrated LLM that generates Dutch-language discharge summaries in real-time and without physician post-editing across multiple departments.

To support interpretation of the findings in relation to real-world deployment, it is important to distinguish between the study-specific system configuration and the current live EHR implementation. Although the LLM was technically integrated within the EHR environment at the time of evaluation, it was configured to operate in a fully automated, non-editable mode for the purposes of this validation study. This configuration differs from the current clinical deployment, in which LLM-generated discharge summaries are presented to physicians as editable drafts. By explicitly evaluating the system in a fully automated setting, this study addresses a foundational question regarding whether the model can generate discharge summaries of sufficient clinical quality prior to human intervention. The implications of transitioning from this controlled evaluation setting to interactive, clinician-edited workflows are therefore relevant for generalisability and are discussed in the context of future implementation and workflow studies within our research team.

While AI-assisted workflows in which clinicians review and edit LLM-generated drafts represent an important real-world use case, the present study intentionally focused on evaluating the intrinsic quality of LLM-generated discharge summaries prior to human intervention. Including physician-edited outputs would have introduced substantial variability related to individual writing style, preferences for length or detail, and formatting choices, thereby confounding model-level strengths and deficiencies. Establishing that the model produces clinically acceptable summaries on its own is a prerequisite before evaluation of editing behaviour and workflow impact. The implications of AI-assisted use, including physician post-editing and efficiency outcomes, are therefore beyond the scope of this analysis and are currently being addressed in a separate follow-up study within our research team.

Most departments showed consistent performance, despite the use of a single prompt across diverse clinical specialties. This suggests prompt robustness and generalisability, though more tailored, department-specific prompting may further improve outcomes.^14^ Only a small fraction (6.8%) of summaries received low completeness scores. These cases reflected omissions rather than fabrications, indicating a need for prompt refinement but not raising concern for hallucinations. Similar findings were reported by Zaretsky et al. and Williams et al., both of whom noted that while LLM-generated summaries were generally accurate and coherent, occasional omissions required human oversight.^11^^,^^12^

Hallucinations represent a critical safety concern in clinical text generation. In this evaluation, no hallucinated or clinically implausible information was identified by the clinician raters. While hallucinations were not scored as a separate domain, raters were able to provide free-text comments during the evaluation, including noting hallucinated content. Safety-relevant omissions were instead reflected in reduced completeness scores and were further examined through an in-depth analysis of low-scoring summaries.

The integration of generative AI into discharge workflows may meaningfully reduce the time clinicians spend on documentation. Although we intended to measure time-to-completion, this metric was not yet available within the EHR environment during the study period. Nevertheless, capturing time metrics remains an important direction for future implementation research, as it would enable more precise estimation of productivity, particularly when AI-generated drafts are reviewed and edited by clinicians rather than written from scratch.

A key challenge in adopting LLMs for clinical documentation is the lack of robust evaluation standards. Traditional Natural Language Processing (NLP) metrics like BLEU and ROUGE often fail to capture factual accuracy or clinical relevance, often assigning high scores to summaries that omit key findings or include incorrect information.^15^ Multiple studies have shown that these metrics poorly correlate with expert clinical judgement and lack the nuance required to evaluate complex, domain-specific content in medical texts.^15^, ^16^, ^17^ To enable structured, reproducible, and clinically grounded evaluation, a Dutch Universal NLP Evaluation System (DUNES) is being developed in this research group to enable a reliable benchmark for automatically assessing AI-generated clinical texts.

Several limitations should be noted. First, the study was conducted at a single Dutch academic hospital and limited to Dutch-language summaries, which may affect generalisability. In addition, although the study draws from a large pool of hospital discharges, a sub analysis comparing the included cases with the full population of discharges from the same period could not be performed. As a result, it cannot be determined whether certain departments were over- or under-represented among finalised discharge summaries, and potential selection bias cannot be fully excluded. Although discharge summaries from 16 departments were included, department-level analyses were limited to departments with at least two cases, as single-case representation does not allow meaningful subgroup comparison. Future validation studies should therefore include larger samples from currently underrepresented departments to enable more robust assessment of department-specific performance. Second, evaluation scores were clustered near the upper end of the Likert scale, indicating a ceiling effect. This may have limited the sensitivity of the instrument to detect subtle differences between systems and affect comparability with studies using different evaluation frameworks. Third, this evaluation was limited to a single vendor-supported, EHR-integrated LLM, with model selection constrained by the in-hospital system. As alternative models were not configurable, direct comparison across vendors and architectures was not feasible, limiting generalisability but reflecting real-world deployment conditions. In addition, the evaluated LLM was embedded in an EHR-integrated system with provider-managed configuration, limiting transparency into the underlying prompt and restricting the ability to adapt prompt design or generation parameters. Fourth, this study did not evaluate physician-edited AI outputs, and therefore does not capture how clinician post-editing may influence final documentation quality in routine deployment. Furthermore, raters reviewed summaries without access to full EHR data; however, this mimics how summaries are often used in practice, such as in primary care. Moreover, evaluations were sometimes conducted by clinicians outside the summary’s specialty, which may introduce bias but also reflects real-world handovers. Finally, the use of LLM-generated summaries may reduce opportunities for junior doctors to practice discharge documentation, which can support clinical reasoning and communication skills. However, this learning is typically informal and varies across departments, while more structured learning occurs through handovers, case discussions, and supervision. Implementation should therefore consider how to preserve educational value while reducing administrative burden.

In conclusion, this study shows that discharge summaries generated by an EHR-integrated language model can meet clinical quality standards, with ratings comparable to physician-written documentation across multiple specialties. While clinical validation and human oversight remain essential, these findings support the potential of generative AI to reduce documentation burden and support safe and timely care transitions.

As LLM applications expand exponentially, continuous monitoring of model performance after implementation is crucial to identify and address potential errors that may go unnoticed by clinicians. The next step is to prospectively evaluate the impact on workflow, time metrics, and long-term adoption. Further work may explore model robustness by assessing how variations in key patient characteristics, such as age or sex, influence generated discharge summaries. To enable safe and widely adoptable use of such tools, clinically grounded evaluation frameworks, such as the one currently under development, will be critical to ensure sustained quality and build trust in clinical practice.

## Contributors

Conceptualisation: TM, TN, AHH, TPL, RCS & CBR, Methodology: TM, TN, AHH, RW, JND, RCS & CBR, Data curation: TM, TN, KK, RW & CBR, Formal analysis: TM & TN, Supervision: TPL, JND, RCS & CBR, Writing, original draft: TM & CBR.

Writing, review & editing: TM, TN, AHH, TPL, KK, RW, JND, RCS & CBR, Data verification: TM & CBR. TM and CBR accessed and verified the underlying data. All authors had full access to the data reported in the study. All authors read and approved the final version of the manuscript. Members of the Applied Artificial Intelligence in Healthcare Consortium contributed as a sounding board during study development, provided feedback on system functionality, and participated as testers and evaluators during different phases of the project. Consortium members were not involved in the formal statistical analyses or manuscript drafting unless explicitly listed as authors above.

## Data sharing statement

Individual participant discharge summaries will not be shared due to patient confidentiality and institutional data protection regulations. The exact prompt text cannot be shared due to proprietary vendor restrictions. De-identified evaluation data underlying the results reported in this Article and the study protocol may be made available upon reasonable request to the corresponding author, subject to approval by the study team and the UMCG Data Access Committee and completion of a data access agreement.

## Declaration of interests

Authors declare that they have no competing interests. The consortium involved in this study did not receive external funding.

## Appendix A Supplementary data

Supplementary Figs. S1–S3 and Tables S1–S6

Members of the AAIH Consortium
