# Supplementary material for: Assessing the quality of AI-generated and physician-written discharge summaries: evaluation of an EHR-integrated tool in a Dutch academic hospital
Source: eBioMedicine. 2026 Apr 9;127:106247. doi: 10.1016/j.ebiom.2026.106247 (PMC13091380; doi:10.1016/j.ebiom.2026.106247)
Supplement: Supplementary Figs. S1–S3 and Tables S1–S6 [file mmc1.docx]

Supplementary material

Contents

[Distribution of discharge letters across departments 1](#_Toc217316558)

[Length of discharge letters 3](#_Toc217316559)

[General descriptives 3](#_Toc217316560)

[Descriptives per department 5](#_Toc217316561)

[Examples of discharge letters 6](#_Toc217316562)

[Original instructions for scoring 7](#_Toc217316563)

# Distribution of discharge letters across departments

| **Department** | **Frequency** | **Percent** | **Valid Percent** | **Cumulative Percent** |
| --- | --- | --- | --- | --- |
| Anesthesiology | 1 | 0.3 | 0.3 | 0.3 |
| Cardiology | 19 | 6.5 | 6.5 | 6.8 |
| Cardiothoracic Surgery | 1 | 0.3 | 0.3 | 7.1 |
| Dentistry | 3 | 1.0 | 1.0 | 8.1 |
| Emergency Medicine | 15 | 5.1 | 5.1 | 13.2 |
| Gastroenterology and Hepatology | 20 | 6.8 | 6.8 | 20.0 |
| General Surgery | 42 | 14.4 | 14.4 | 34.4 |
| Internal Medicine | 61 | 20.9 | 20.9 | 55.3 |
| Neurology | 27 | 9.2 | 9.2 | 64.5 |
| Obstetrics and Gynecology | 12 | 4.1 | 4.1 | 68.6 |
| Orthopedics | 29 | 9.9 | 9.9 | 78.5 |
| Otorhinolaryngology (ENT) | 22 | 7.5 | 7.5 | 86.0 |
| Pediatrics | 29 | 9.9 | 9.9 | 95.9 |
| Psychiatry | 1 | 0.3 | 0.3 | 96.2 |
| Pulmonology | 9 | 3.1 | 3.1 | 99.3 |
| Rehabilitation Medicine | 1 | 0.3 | 0.3 | 99.6 |
| **Total** | **292** | **100.0** | **100.0** |  |

Table S1, Distribution of discharge letters across departments.

This table shows the number and relative proportion of discharge summaries included per clinical department. Percentages are calculated relative to the total study sample (n = 292)

Figure S1, Distribution of discharge letters per department.

Bar chart visualizing the distribution of included discharge letters across clinical departments.

# Length of discharge letters

## General descriptives

| **Summary type** | **n** | **Mean** | **SD** | **Minimum** | **Maximum** |
| --- | --- | --- | --- | --- | --- |
| Physician-written | 292 | 204.13 | 113.54 | 40 | 966 |
| LLM-generated | 292 | 123.63 | 18.53 | 68 | 176 |

Table S2, Average wordcount physician-written and LLM-generated discharge letters.

| **Statistic** | **Physician-written** | **LLM-generated** |
| --- | --- | --- |
| Mean | 204.13 | 123.63 |
| Median | 178.00 | 123.00 |
| 5% trimmed mean | 193.52 | 123.99 |
| Standard deviation | 113.54 | 18.53 |
| Variance | 12,890.27 | 343.29 |
| Interquartile range (IQR) | 132 | 21 |
| Range | 926 | 108 |
| Skewness (SE) | 2.43 (0.14) | −0.24 (0.14) |
| Kurtosis (SE) | 10.77 (0.28) | 0.45 (0.28) |

Table S3, Distribution characteristics of word counts.

Percentiles

| **Percentile** | **Physician-written** | **LLM-generated** |
| --- | --- | --- |
| 25th percentile | 131.25 | 113.00 |
| 50th percentile (median) | 178.00 | 123.00 |
| 75th percentile | 263.00 | 134.00 |

Table S4, Percentiles of word count distributions

95% confidence interval for the mean

| **Summary type** | **Lower bound** | **Upper bound** |
| --- | --- | --- |
| Physician-written | 191.06 | 217.21 |
| LLM-generated | 121.50 | 125.77 |

Table S5, 95% Confidence intervals for mean word count


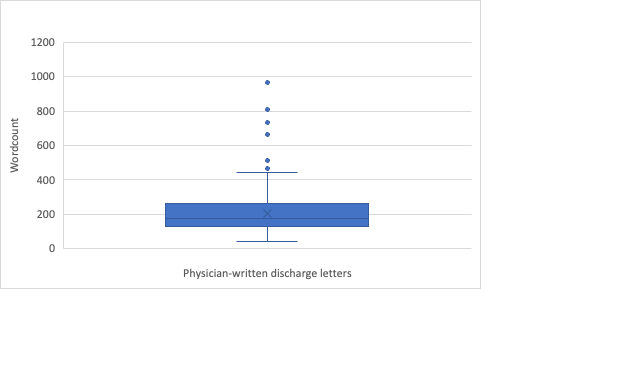


Figure S2, Box plot showing the distribution of word counts of physician-written discharge letters.


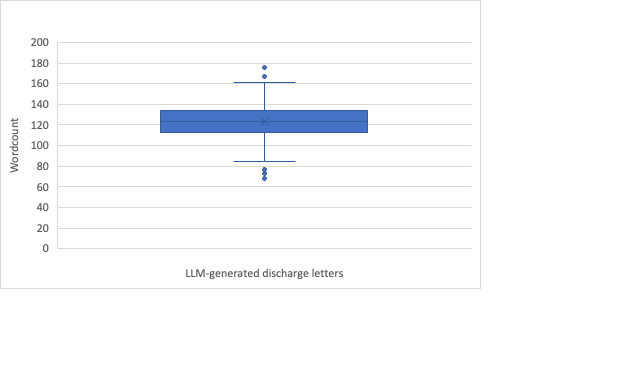


Figure S3, Box plot showing the distribution of word counts of LLM-generated discharge letters.

## Descriptives per department

| **Department** | **n** | **Physician-written summaries, mean (SD)** | **Min–Max** | **LLM-generated summaries, mean (SD)** | **Min–Max** |
| --- | --- | --- | --- | --- | --- |
| Anesthesiology | 1 | 341.00 (–) | 341–341 | 123.00 (–) | 123–123 |
| Cardiology | 19 | 198.26 (80.59) | 113–443 | 119.95 (22.94) | 68–160 |
| Cardiothoracic Surgery | 1 | 145.00 (–) | 145–145 | 131.00 (–) | 131–131 |
| Dentistry | 3 | 165.00 (48.59) | 109–196 | 99.00 (26.29) | 80–129 |
| Emergency Medicine | 15 | 168.40 (72.66) | 87–314 | 109.93 (18.53) | 73–137 |
| Gastroenterology and Hepatology | 20 | 267.30 (142.49) | 86–737 | 122.00 (15.32) | 98–154 |
| General Surgery | 42 | 242.07 (119.05) | 40–666 | 123.26 (20.27) | 74–161 |
| Internal Medicine | 61 | 212.46 (93.37) | 62–512 | 125.93 (11.56) | 88–152 |
| Neurology | 27 | 202.67 (96.74) | 56–378 | 128.85 (17.93) | 100–167 |
| Obstetrics and Gynecology | 12 | 175.67 (53.70) | 81–270 | 111.33 (15.83) | 86–145 |
| Orthopedics | 29 | 142.83 (41.41) | 68–213 | 124.83 (22.75) | 76–170 |
| Otorhinolaryngology (ENT) | 22 | 180.27 (93.13) | 70–416 | 123.55 (16.95) | 80–161 |
| Pediatrics | 29 | 181.48 (167.53) | 83–966 | 126.21 (18.73) | 92–159 |
| Psychiatry | 1 | 174.00 (–) | 174–174 | 121.00 (–) | 121–121 |
| Pulmonology | 9 | 293.78 (209.54) | 74–811 | 139.11 (19.35) | 107–176 |
| Rehabilitation Medicine | 1 | 91.00 (–) | 91–91 | 137.00 (–) | 137–137 |
| **Total** | **292** | **204.13 (113.54)** | **40–966** | **123.63 (18.53)** | **68–176** |

Table S6, Word count per department for physician-written and LLM-generated discharge summaries

# Examples of discharge letters

The patient was admitted to the department of Gastroenterology and Hepatology of UMCG.
Reason for admission: colonoscopy
Alcohol use: Never. Drug use: Never.

History at admission:
Is very talkative. Generally doing quite well. Has a smooth liquid diet, with that it goes well. Occasionally cramps for several hours, then also spontaneously subsides. Stool always loose, does not use Movicolon for this. Not nauseous or vomiting. Stopped with ferro fumarate a week ago. No fever or chills in the past period, has not been ill. Talks extensively about delirium 2 years ago, happy to have recovered well from it. Now receives home care. Mobilizes with walker, this goes well.

Conclusion: Again signs of (mucosal) active inflammation at the site of the neoterminal ileum with stenosis and prestenotic dilatation. The degree of inflammation is less pronounced than on [date]. Length of affected segment approximately 7 cm.

Endoscopy:
Colonoscopy [date]
Smooth introduction up to the ileocolic anastomosis, which is just not passable. The anastomosis is dilated with 12–15 mm CRE balloon and then 15 to 18 mm. After this, passage is possible. In the neoterminal ileum no stenosis, no ulcers, some redness. In the colon especially right-sided some redness. The bowel is well cleansed. No diverticula seen. Rectum viewed during inversion. No internal hemorrhoids.

Conclusion: Stenosis ileocolic anastomosis, dilated up to 18 mm with CRE

Discussion:
The above-mentioned 78-year-old female patient with Crohn’s disease of the terminal ileum and colon, status after ileocecal resection (1980) and multiple times a dilatation of the neoterminal ileum, was admitted on 13-4 for clinical preparation for colonoscopy. This proceeded without problems and on [date] the colonoscopy takes place during which the stenosis at the level of the ileocolic anastomosis was dilated. Afterwards patient could be discharged home in good clinical condition.

Conclusions:
Dilatation of stenosis ileocolic anastomosis

Follow-up plan summarized:
– Follow-up already scheduled for [date] with [name doctor]
– Taper budesonide after dilatation
 (once daily 6 mg for 30 days, then once daily 3 mg for 30 days)

Treatment limitations during admission: full code

During this admission a complication occurred and this complication was reported for the respective complication discussion: no

Figure S4, Example of PHI-free physician-written discharge letter

A patient with Crohn’s disease and chronic renal insufficiency was admitted for the preparation of a colonoscopy because of symptomatic stenosis of the terminal ileum and the ileocolic anastomosis.
The colonoscopy on [date] showed a stenosis at the ileocolic anastomosis, which was dilated up to 18 mm with a CRE balloon, after which passage was possible.
During the admission, ferro fumarate was stopped.
The patient felt well after the procedure and had no pain.
There were no complications during the therapeutic intervention.
Her medication was not adjusted, except for tapering of budesonide after the dilatation.
The patient was discharged the same day and would be picked up by family.

Figure S5, Example of PHI-free LLM-generated discharge letter

Original instructions for scoring

***Assessment instructions for validation Drafted Hospital Course***

*Dear reviewer,*

*Thank you for participating in this evaluation. For each patient, you will be presented with two discharge summaries: Letter A and Letter B. One summary was written by a physician, and the other was generated by an AI system. You will not be informed in advance which summary was AI-generated or physician-written.*

1. ***For each summary separately (i.e., both Letter A and Letter B), please evaluate three domains: completeness, correctness, and conciseness. Each domain should be scored on a scale from 1 to 5, based on the evaluation criteria provided below.***

***Completeness Grading Scale (1–5)***

*Does it have all essential information?*

*1 -****Severely Incomplete****: Missing substantial portions of original content, key facts omitted, possible fabrication.  
2 -****Significantly Incomplete****: Captures some relevant information but omits major details, distorting context.  
3 -****Moderately Complete****: Most essential information included but with some omissions or minor irrelevant details.  
4 -****Mostly Complete****: All essential details present with minor omissions or redundancies.  
5 -****Fully Complete****: Perfectly mirrors original content with no fabrications.*

***Correctness Grading Scale (1–5)***

*Is the information accurate, ie no false information, misinterpretations, or hallucinations?*

*1 -****Severely Incorrect****: Riddled with factual inaccuracies, misrepresented data, or fabrications.  
2 -****Significantly Incorrect****: Contains substantial inaccuracies or misinterpretations of key details.  
3 -****Partially Correct****: Mostly accurate but contains minor errors or misinterpretations.  
4 -****Mostly Correct****: Nearly all key facts are accurate with minor, non-impactful errors.  
5 -****Fully Correct****: Entirely accurate, preserving essential facts and meaning with no errors.*

***Conciseness Grading Scale (1–5)***

*Is the information concise, without unnecessary details?*

*1 -****Severely Wordy or Sparse****: Excessively long with redundant details or too brief to be useful.  
2 -****Poorly Balanced****: Contains irrelevant information or omits critical details.  
3 -****Moderately Concise****: Removes much unnecessary detail but retains minor redundancies.  
4 -****Mostly Concise****: Well-balanced with minimal unnecessary details.  
5 -****Exceptionally Concise****: Retains only the most relevant information with optimal clarity.*

1. ***Trust***

*Please answer the following question with* ***yes*** *or* ***no****.*

“Would you trust this discharge summary for routine clinical use?”

1. ***Additional comments***

*If you have additional remarks about the text or notice any issues, you may note these in the free-text field (for example, hallucinations or clinically relevant omissions).*

*Please note: try to assess each summary independently and refrain from speculating about the authorship.*

*Thank you for your contribution!*

Figure S6, original assessment instructions

**Decide AI checklist**

| **Item No** | **Theme** | **Recommendation** | **Reported on page** |
| --- | --- | --- | --- |
| 1 | Title | Identify the study as early clinical evaluation of an AI-based decision support system, specifying the problem addressed | Title; Abstract |
| I | Abstract | Structured summary including intended use, algorithm type, setting, N patients/users, outcomes, safety endpoints, human factors, main results, conclusions | Abstract |
| 2 | Intended use | a) Targeted problem, standard practice, intended population; b) intended users, integration, intended impact | Introduction; Methods (Model Prompting and Generation) |
| II | Objectives | State study objectives | Introduction (final paragraph) |
| III | Research governance | Protocol/registration and ethics approval | Methods (Ethical Considerations) |
| 3 | Participants | a) Patient recruitment/inclusion/exclusion and sample size decision; b) user recruitment; c) user familiarisation/training | Methods (Data Sources and Case Selection; Evaluation Framework) |
| 4 | AI system | a) System description/version/algorithm & training cutoff; b) inputs, acquisition, preprocessing/missing data; c) outputs & presentation | Methods (Model Prompting and Generation) |
| 5 | Implementation | a) Evaluation setting; b) workflow/care pathway and how final decision reached | Methods (Study Design and Setting; Model Prompting and Generation) |
| IV | Outcomes | Specify primary and secondary outcomes | Methods (Evaluation Framework) |
| 6 | Safety and errors | a) Define errors/malfunctions; b) describe harm identification/minimisation | N/A (research-only configuration; no clinical deployment/patient exposure) |
| 7 | Human factors | Human factors tools/methods/frameworks used | Methods (Evaluation Framework; trust assessment; blinding) |
| V | Analysis | Statistical methods including subgroup analyses | Methods (Statistical Analysis) |
| 8 | Ethics | Ethics-related methodology (e.g., fairness) | Methods (Ethical Considerations) |
| VI | Patient involvement | State patient involvement | N/A (no patient involvement) |
| 9 | Participants (Results) | a) Baseline patient characteristics + input missingness; b) baseline user characteristics | Results (Study Sample and Distribution); Methods (Evaluation Framework) |
| 10 | Implementation (Results) | a) user exposure/adherence; b) workflow changes | Results; N/A (no workflow change) |
| VII | Main results | Report prespecified outcomes | Results |
| VIII | Subgroups analysis | Report subgroup differences | Results (Subgroup Analysis by Department) |
| 11 | Modifications | Changes to AI system/hardware during study | Methods (Model Prompting and Generation) |
| 12 | Human-computer agreement | User agreement with AI recommendations | N/A / Limited applicability (no decision recommendations; paired ratings reported in Results) |
| 13 | Safety and errors | a) errors/malfunctions; b) harms | N/A (no clinical deployment/patient exposure) |
| 14 | Human factors | a) usability; b) learning curves | Not formally assessed; discussed as limitation (Discussion) |
| 15 | Support for intended use | Discuss whether results support intended use | Discussion |
| 16 | Safety and errors | Discuss safety profile and mitigation | Discussion (hallucination discussion; limitations) |
| IX | Strengths and limitations | Discuss strengths/limitations | Discussion (Limitations) |
| 17 | Data availability | Data/code availability | Data Sharing Statement |
| X | Conflicts of interest | Conflicts/funding/vendor role | Declaration of Interests; Methods (Role of Funders) |
