# Supplementary material for: Assessing the quality of AI-generated and physician-written discharge summaries: evaluation of an EHR-integrated tool in a Dutch academic hospital
Source: eBioMedicine. 2026 Apr 9;127:106247. doi: 10.1016/j.ebiom.2026.106247 (PMC13091380; doi:10.1016/j.ebiom.2026.106247)
Supplement: Members of the AAIH Consortium [file mmc2.docx]

**Members of the Applied Artificial Intelligence in Healthcare Consortium**

| **First names** | **Surname** |
| --- | --- |
| M | Aalderink |
| R | Van den Berg |
| M T P | Besouw |
| A V | Biere |
| F A J A | Bodewes |
| A L | Boerboom |
| M A J | Borgdorff |
| M H | De Borst |
| M | Bouhuys |
| B R | Brandsema |
| G H | Bultema |
| M J | Crop |
| H P J | Van der Doef |
| J W J | Donkers |
| J M | Douwes |
| R A | Feijen |
| F | Fontanella |
| B | Foreman |
| V | Gracchi |
| I | De Groot |
| G B | Halmos |
| A A | Van Heerwaarde |
| F | Van den Heuvel |
| C | Holzhauer |
| F F A | IJpma |
| E | Kersten |
| R J H | Knoef |
| M C A | Kramer |
| S | Krishnapillai |
| M | Labberté |
| J M | Lammers |
| L B | De Langen |
| E | Lensen |
| W S | Lexmond |
| E T | Liem |
| E | Loeffen |
| J | Lorius |
| C | Lubout |
| J | Ludwig-Roukema |
| S | Luiten |
| D | Meijering |
| C | Out |
| S | Palthe |
| M T R | Roofthooft |
| R | Scheenstra |
| R S B H | Schreuder |
| M L | Schrijvers |
| P F | Sinnige |
| W J | Van Veen |
| C A | Te Velde-Keyzer |
| K T | Verbruggen |
| M | Verheijen |
| F P J | Vernimmen |
| J | De Vries |
| W | De Weerd |
| C L | Welsink |
| J E J | Woolderink |
| A T | Zwart |
